# Quantifying Staff Preferences for Workforce Retention Strategies in Remote Australian Primary Health Care Services

**DOI:** 10.1111/ajr.70170

**Published:** 2026-03-27

**Authors:** Nanda Kaji Budhathoki, John S. Humphreys, Supriya Mathew, Leander Menezes, Mark Ramjan, Donna‐Maree Stephens, Karrina DeMasi, Yuejen Zhao, Kristal Lawrence, Deborah Russell

**Affiliations:** ^1^ Menzies School of Health Research Charles Darwin University Alice Springs Northern Territory Australia; ^2^ Monash Rural Health Monash University Bendigo Victoria Australia; ^3^ Pintupi Homelands Health Service Aboriginal Corporation Kintore Northern Territory Australia; ^4^ Northern Territory Department of Health Darwin Northern Territory Australia; ^5^ DMS Research & Evaluation Consulting Darwin Northern Territory Australia; ^6^ South Australia Health, Department for Health and Wellbeing Adelaide South Australia Australia

**Keywords:** best worst scaling, health workers, preferences, remote and rural, retention

## Abstract

**Aim:**

To outline key criteria when choosing how to measure remote area primary health care workers' retention preferences and compare six different methodological approaches against these criteria.

**Context:**

Australians living in remote communities experience poorer health outcomes. Many health services serving remote populations have persistent workforce shortages, high staff turnover and short retention which adversely affect access to timely, appropriate health care, care continuity and cultural appropriateness of care. Improved understanding of staff preferences for different retention initiatives could inform initiatives to improve workforce stability.

**Approach:**

Measuring the preferences of remote primary health care staff is complex. Key criteria to guide methodology choice include: i) logistical considerations related to geographical remoteness, ii) diversity of primary care staff, iii) minimising participants' cognitive load and time required for data collection, iv) ability to quantify preferences, v) appropriateness of methodologies for staff from different socio‐cultural backgrounds, vi) minimising bias, and vii) making trade‐offs given limited resources for implementing retention incentives. Six different methodological approaches are compared: Rating, Ranking, Best Worst Scaling (BWS) Object Case, BWS Profile Case, BWS Multi‐profile Case and conventional Discrete Choice Experiments (DCE).

**Conclusion:**

The comparison of different methodologies reveals the advantages of using a BWS Object Case methodological approach for measuring primary health care service workers' preferences in remote primary health care settings. Importantly, this paper demonstrates how the quantified results from BWS object case analysis can be translated into actionable retention initiatives designed to retain remote area health workers, given ongoing budget constraints and the need to ensure cultural appropriateness.

What is already known on this subject?

- Remote Australian health services have been persistently impacted by high turnover of health care staff.
- Stated preference survey methods have only been occasionally applied in health research in high income countries to understand health care workers' preferences in remote settings.
- Different methodological approaches have a range of strengths and weaknesses which may make them more or less suitable for use in remote settings.

What does this study add?

- This study compares a range of methodological approaches that could be used to measure remote primary health care service staff members' preferences.
- A rationale is provided for using a Best‐Worst Scaling Object Case methodological approach for measuring primary health care service staff members' preferences in remote Aboriginal health care settings.

## 1 Introduction

Populations residing in remote areas generally experience poorer health outcomes than urban populations. Global estimates show more than half the population living in remote and rural regions have inadequate access to essential health care facilities, leading to adverse impacts on health outcomes [1]. Difficulties with recruitment and retention of health professionals in remote areas result in inadequate supply of suitably skilled health care providers relative to high population health need [2], which is a major contributor to poorer access to quality health care [3].

Poor retention of remote health providers exacerbates workforce shortages and results in high financial burdens on health services [4]. Poor retention also reduces continuity of patient care which can lead to patient disengagement with their health care, delays in seeking care, and negative effects on patient experiences of culturally safe care [5]. Elsewhere, lower continuity of care of primary health care (PHC) providers has been associated with increased mortality amongst vulnerable populations [6].

Optimising the retention of staff working in PHC services in remote Australia is thus critically important. Previous research has quantified existing high levels of staff turnover and high use of temporary staff [7, 8, 9]. A wide range of strategies spanning the workforce education and training continuum are needed to address these issues, including preferential student selection and targeted training; initiatives targeting workplace arrangements and personal supports; financial incentives and regulatory interventions [10, 11, 12].

One recent systematic review synthesised quantitative studies of the effectiveness of rural and remote retention interventions [13]. While it demonstrated significant variation in their effectiveness, it also highlighted how little is known about which workforce retention strategies are preferred by different groups of employees working in remote health services. Jaskiewicz, Deussom et al. [14] developed a rapid retention survey toolkit using conventional Discrete Choice Experiments (DCEs) to determine preferred bundles of incentives to attract and retain health workers. While there are several examples of DCEs being used in rural Australia to ascertain General Practitioner (GP) perceptions of the relative importance of different retention factors [15] and GP preferences for different attributes of retention incentives [16], the rapid retention survey toolkit has, to the authors' knowledge, not been applied in Australian remote settings.

Because remote health care service issues differ from rural in many ways [17], addressing this research gap could better inform how remote PHC services design and implement effective retention strategies for staff members. The aim of this article is to outline and justify approaches to strengthening the evidence about the preferences of different groups of remote primary health service staff for different retention strategy features, as the basis for developing appropriate and effective retention policies and bundles.

## 2 The Conceptual Basis Underpinning Choice of the Data Collection Instrument

Health worker retention is influenced by a complex interplay of individual, social and professional factors, which vary across countries, geographical regions, professional contexts, and career stages, as well as across cultural, social, and economic environmental factors [15, 18]. In order to maximise the effectiveness of retention strategies it is necessary to target not just a single mutable factor, but instead to bundle together a range of strategies which target the most important retention issues [9, 13, 18, 19]. To do this, proposed retention policy initiatives must accord with workforce preferences. The following section outlines key criteria and discusses numerous factors that should be taken into consideration when choosing an appropriate methodology for collecting empirical data on the preferences of remote health workers.

## 3 Key Criteria Underpinning the Choice of Methodology

Several considerations and constraints (including logistical, cultural, methodological, and resource issues) affect the choice of methodology for measuring workforce retention preferences of people working in Australian remote PHC services.

Firstly, remote PHC services are geographically scattered across remote Australia. This means that conducting only face‐to‐face interviews is logistically difficult and expensive due to vast distances, unsealed roads, and often extreme weather conditions. Remote community accommodation shortages, the need for remote community travel permits, and restrictions to travel during cultural ceremony periods also limit timely access to some very remote sites by researchers. Limited resourcing often tied to grants necessitates that as much data as possible are collected digitally (such as using an online survey, phone, or online interviews).

Secondly, remote PHC services generally offer comprehensive care (including pregnancy care and child health through to aged care and palliative care, in addition to health promotion, disease prevention, treatment, management and acute care) and employ a wide range of different personnel groups, including Remote Area Nurses and midwives, Aboriginal and Torres Strait Islander Health Practitioners and Health Workers, GPs, administrative and various support staff including drivers, cleaners, gardeners, personal care assistants and maintenance staff. Workplace retention issues commonly differ in importance across the broad range of clinical and non‐clinical staff and also between local and non‐local staff employed by remote PHC services. In small remote teams, where every staff member plays a pivotal role, understanding the preferences for a wide range of retention initiatives of all staff groups is essential to inform targeted retention strategies.

Thirdly, staff working in remote PHC services usually experience heavy workloads and work long hours, often in an under‐resourced and stressful environment. It is therefore important to ensure that data collection is not onerous and does not impinge excessively on their work activities. A short and cognitively simple online survey provides staff with flexibility to complete the survey at a time that is convenient to them and limits cognitive burden.

Fourthly, it is important to quantify the relative importance of each attribute of a retention initiative as this increases the utility of the findings for remote PHC services and policy makers compared with just rankings. Ranking of retention factors only enables policy makers to know that X is preferred over Y, but not by how much or whether the difference is significant or not. Research approaches which quantify differences between preferences provide more information to inform policy strategies.

Fifthly, in order to have sufficient power to detect important differences between groups, it is important to collect a sufficient number of responses from each group. Some groups of staff employed by remote PHC services may have low English language literacy (as English may not be their first language) or face substantial additional barriers to completing an online survey in English (such as limited digital technology skills, limited digital connectivity, lack of digital device access, and/or cultural preferences for alternative means of providing information for research). It is therefore important to ensure appropriate data collection methodologies (e.g., yarning circles with local Aboriginal and Torres Strait Islander staff) are implemented in order to obtain a sufficient sample from such employee groups. This may require research teams to engage and train local Aboriginal and/or Torres Strait Islander community‐based researchers to lead this aspect of data collection using culturally appropriate ways of data collection.

Sixthly, the method chosen should limit sources of bias such as agreement (acquiescence) bias, social desirability, and extreme response bias [20, 21].

Lastly, a method should be used that drives participants to make trade‐offs between different options while selecting their preferences [20]. When PHC staff respondents choose one attribute of a retention initiative over another, it facilitates increased understanding of what they value. As remote PHC services operate with limited resources, it is useful if the method informs understanding of opportunity costs, or what attribute of a retention initiative different staff members are willing to forego or trade off.

## 4 To What Extent Do Different Methodologies Meet These Criteria

**FIGURE 1:**
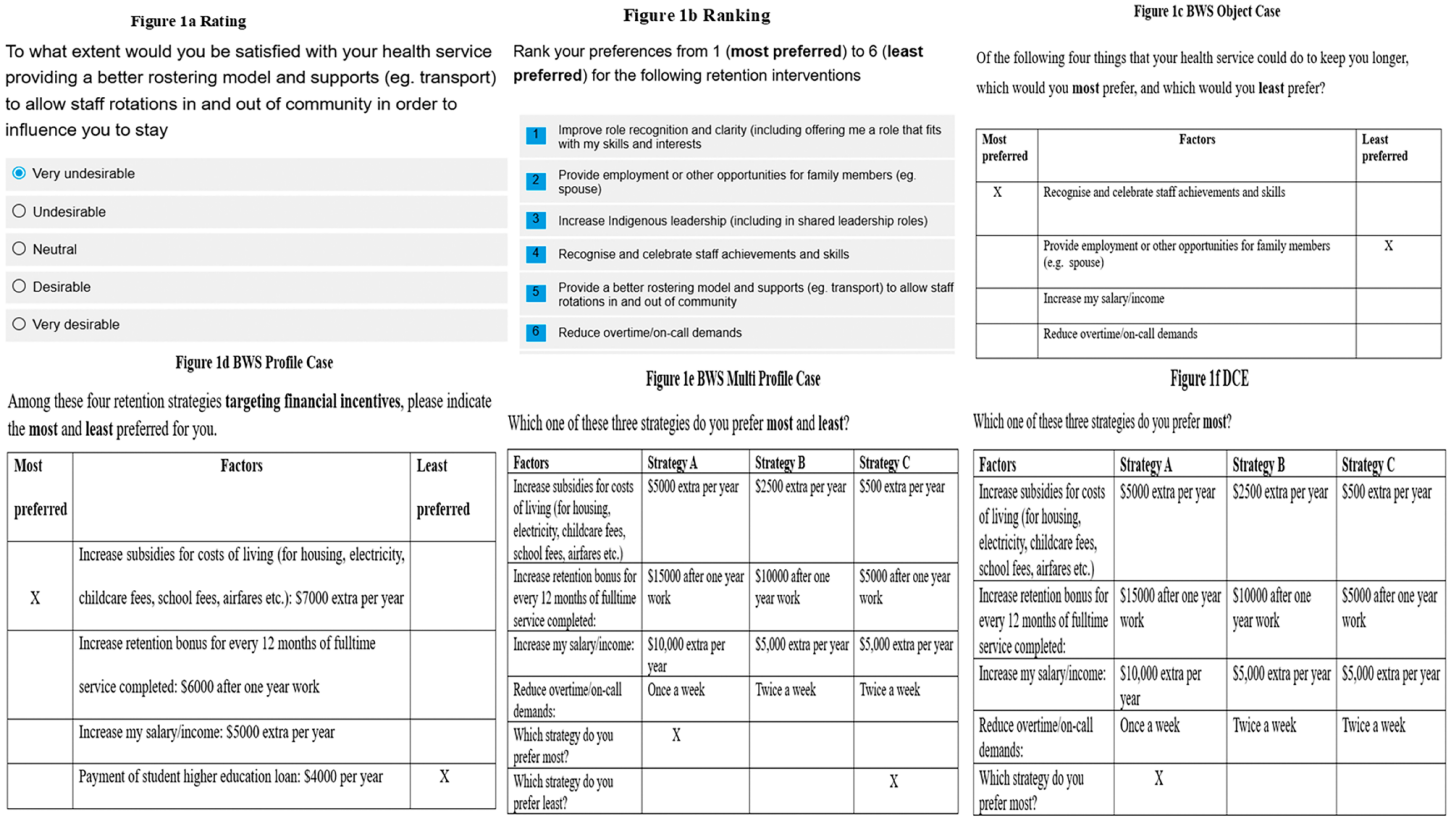
Examples illustrating different methods for eliciting preferences.

In this section, a number of different methodologies for assessing staff preferences will be considered and compared: Rating, Ranking, Best Worst Scaling (BWS) Object Case (Case 1), BWS Profile Case (Case 2), BWS Multi‐profile Case, and conventional DCE [20, 22, 23]. Examples of a survey question using each of these methodologies are shown in Figure 1.

These methodologies are assessed against the different criteria mentioned above (Table 1). Rating and ranking methodologies are easy to understand and implement but offer limited predictive and discrimination power. BWS Object Case has greater predictive and discriminatory power and is also simple and easy to implement with a long list of attributes. In contrast, BWS Profile, multi‐profile, and conventional DCE aren't as simple and easy for respondents and don't handle long lists of attributes as well as BWS Object Case. These differences are explained in more detail in the following section.

**TABLE 1:** Comparison of different methodological approaches to understanding remote PHC staff preferences.

Criteria	Rating	Ranking	BWS Object Case	BWS Profile Case	BWS Multi‐profile Case	Conventional DCE
Structure	Respondents rate items or their attributes using a Likert scale (e.g., 5 levels: very undesirable to very desirable)	Respondents rank items or their attributes in increasing or decreasing order of preference	Respondents choose their single most and single least preferred choices from a short list of items (or ‘objects’)	Respondents choose their single most and single least preferred choices from a short list of attribute levels within one profile.	Three or more profiles are presented in each choice task. Respondents choose their single most and least preferred options from each choice task	Three or more profiles are presented in each choice task. Respondents choose their single most preferred option from each choice task.
Suitability for many attributes or items	May not differentiate between attributes	Poorly handles long list of attributes	Suitable for a long list of items	Possible with limited attributes and levels	Possible but complex – limited attributes and levels	Possible but complex – limited attributes and levels
Cognitive burden	Low. Respondent preferences for each item or their attributes are considered independently of all other items and attributes	Low‐Moderate Increases with overall number of items that are to be ordered in the list	Low	Moderate	High	High. Does not inquire about least preferred options so would require more questions to gather equivalent information compared to BWS Multi‐profile Case
Design complexity	Simple	Simple	Simple	Moderate	High	High
Bias	More prone to social desirability, extreme response and acquiescence biases	More prone to social desirability, extreme response and acquiescence biases	Reduced social desirability, extreme response and acquiescence biases	Reduced social desirability, extreme response and acquiescence biases	Reduced social desirability, extreme response and acquiescence biases	Reduced social desirability, extreme response and acquiescence biases
Ease of implementing with standard survey tools (e.g., Qualtrics)	Yes, straightforward	Yes, straightforward	Yes, straightforward	Possible, but difficult	Possible, but complex	Possible, but complex
Trade off/Discrimination between the items	No trade‐off, and less discriminating between items	No trade‐off, and less discriminating between items	Some trade‐off/discrimination	High trade‐off/discrimination	High trade‐off/discrimination	High trade‐off/discrimination
Strength of relationship	Indicative of order but only differentiates by Likert scale level and multiple items could be accorded the same Likert level. Does not quantify the relative strengths of preferences.	Indicates order but not the relative strengths of preferences	Indicates order and quantifies strength of preference	Indicates order and quantifies strength of preference	Indicates order and quantifies strength of preference	Indicates order and quantifies strength of preference
Predictive power of preferences	Low/weak	Low/weak	High	High	High	High
Interactions taken into account	No	No	No	Yes	Yes	Yes

## 5 Implications for Choice of Methodology

Each of the methodologies have different strengths and weaknesses for ascertaining staff preferences for workforce retention initiatives in remote Australian PHC services. Rating and ranking approaches, while generally being cognitively simple and straightforward to administer, do not quantify preferences. This means that while the order of items is understood, their relative strengths of importance are not known [20, 21]. Rating and ranking approaches also do not require respondents to make trade‐offs between options, whereas economic preference elicitation methodologies (e.g., 1c‐f) do require consideration of trade‐offs [24]. Use of ranking and rating methodologies therefore results in important limitations in the evidence that is subsequently available to inform policy making. Furthermore, rating and ranking scales are also more prone to acquiescence (agreement) bias, social desirability bias (tendency to lie) and extreme response bias compared to economic preference elicitation methodologies. Economic preference elicitation methodologies can also use survey designs such as Balanced Incomplete Block Design. This presents individual respondents with choice sets containing fewer options than the total possible choices while at the same time ensuring that for the overall survey each option is presented a consistent number of times. This design is efficient and reduces cognitive burden on individual respondents, who otherwise may have to answer many more questions.

In the context of remote Australian PHC settings, the BWS Object Case methodology has several advantages over the conventional DCE methodology. To enhance practical understanding, we include a hypothetical scenario illustrating the potential implementation of a BWS survey by a remote PHC service and use of its findings to inform staff retention initiatives (Box 1). BWS methodologies can potentially extract more information by inquiring about respondents' least preferred initiatives, whereas rating, ranking and standard DCE methodologies only inquire about respondents' most preferred initiatives. It also places a significantly lower cognitive burden on respondents compared to BWS Profile Case, BWS Multi‐profile Case and conventional DCE methodologies [21, 24]. It is also able to deal with a longer list of attributes compared to BWS Profile Case, BWS Multi‐profile Case and conventional DCE. This is important if there are many potentially important job‐related retention attributes, as is often the case in remote Australian health services where diverse staff groups are employed.

**BOX 1:**
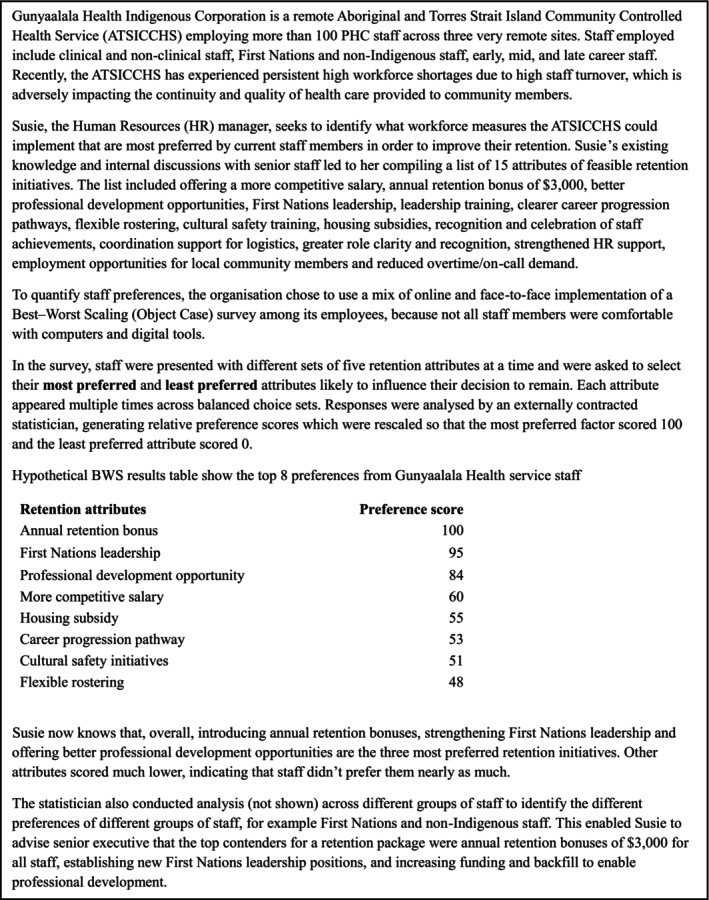
Hypothetical illustration of using BWS Object Case method in a remote PHC organisation.

Significantly, BWS Object Case can be implemented using a combination of in‐person and online methodologies. Researchers may purposively visit remote and very remote health services to provide in‐person support, facilitated by Aboriginal and Torres Strait Islander researchers to enable participation of Aboriginal and Torres Strait Islander staff and culturally respectful administering of the survey. Online recruitment can be supported through partnerships with Aboriginal and Torres Strait Island Community Controlled Health Service (ATSICCHS) and other key remote health service stakeholders. Such groups can play a key role in distributing online survey links or survey materials to their staff and other primary health care staff via their organisational newsletters, internal email lists and social media pages. Survey links and QR codes may also be shared at relevant workshops and remote health conferences. Additionally, online or face‐to‐face surveys could be implemented with the help of trusted community‐based researchers, colleagues, family or friends in remote situations where some staff members may need support to complete a survey.

## 6 Discussion

Based on the key criteria outlined in Table 1, the BWS Object Case methodology exhibits some notable strengths for use in the remote PHC context. It has better predictive and discrimination power than rating and ranking methodologies. It is also simpler and easier to implement than BWS Profile, BWS multi‐profile and conventional DCEs. One of the key criteria for a suitable methodology for remote PHC settings is the ability to handle a longer list of items. The comprehensive nature of PHC services provided in remote settings means that many different staff members are often employed in a broad range of different roles and are therefore likely to have different preferences in relation to retention attributes. Importantly, the BWS Object Case methodology stood apart from other methodologies that could quantify preferences because it can accommodate a longer list of attributes relevant to remote PHC workforce retention. Moreover, the BWS Object Case methodology is relatively easier to understand and less demanding on remote staff members' time and cognitive load than conventional DCE and BWS Multi‐profile Case.

These strengths notwithstanding, BWS Object Case is not without some limitations, particularly in terms of practical implementation of a BWS survey in remote primary PHC settings. Since this is a novel preference elicitation methodology, participants may not be familiar with the survey design, which may result in more incomplete surveys compared with more widely understood ranking and rating methodologies [21]. In remote PHC communities, where English is often a second or third language spoken by local Aboriginal and/or Torres Strait Islander staff and wide variations in digital literacy may also be present, survey implementation may be challenging, and in some communities may be inappropriate. Local cultural guidance should be sought, including to inform BWS survey design, appropriate use of visual aids, plain English language explanations, translations, and to ensure well‐trained culturally respectful survey facilitators. This may require employing Aboriginal and/or Torres Strait Islander community‐based researchers to conduct yarning sessions to elicit preferences of Aboriginal and/or Torres Strait Islander staff members, as an alternative to administering a hard copy or electronic survey. A yarning approach could better support culturally safe engagement and help to clarify survey concepts and retention attributes for participants. It should also be noted that the BWS Object Case methodology does not take interactions between attribute levels into account, thus limiting its ability to capture complex trade‐offs [20, 23]. Instead, calculations reflect the trade‐offs respondents make between attributes (rather than between levels of attributes). Moreover, some participants might be fatigued while making choices from a long list of similar attributes. However, this can be mitigated by balanced incomplete block design whereby each respondent does not get asked the full set of survey questions [25].

## 7 Conclusion

Despite its limitations, the Best Worst Scaling (BWS) Object Case methodology offers an effective, innovative and comprehensive approach for eliciting PHC workforce preferences for retention initiatives. The method can be directly adopted and implemented by medium to large sized remote health services to inform health service‐level workforce retention initiatives. Surveys conducted at regional, jurisdictional and national levels could similarly inform workforce retention policy decisions by respective authorities (e.g., findings from a BWS Object Case survey of GPs working in Australia could be used to inform deliberations by the Australian Government Department of Health, Disability and Ageing's Health Workforce Division on changes to the rural and remote GP retention incentive program). By adopting the BWS Object Case method, policy makers at different levels of the health system will have key information needed to develop more targeted workforce retention bundles that accommodate the different preferences of a diverse range of health care staff, rather than taking a ‘one‐coat‐fits‐all’ approach. In this way, workforce investments and policies aimed at improving workforce retention in remote health care settings can be better designed, implemented and evaluated in terms of both their appropriateness and effectiveness.

## Author Contributions

**Nanda Kaji Budhathoki:** conceptualization, writing – original draft, writing – review and editing, visualization, methodology. **John S. Humphreys:** writing – review and editing, conceptualization, visualization, funding acquisition, mentoring, methodology. **Supriya Mathew:** writing – review and editing, conceptualization, visualization, methodology. **Leander Menezes:** writing – review and editing. **Mark Ramjan:** writing – review and editing. **Donna‐Maree Stephens:** writing – review and editing, funding acquisition. **Karrina DeMasi:** writing – review and editing, funding acquisition. **Yuejen Zhao:** writing – review and editing, funding acquisition. **Kristal Lawrence:** writing – review and editing, project administration. **Deborah Russell:** writing – review and editing, visualization, conceptualization, funding acquisition, supervision, methodology.

## Funding

The authors acknowledge the financial support of the Cooperative Research Centre for Developing Northern Australia (CRCNA), which is part of the Australian Government’s Cooperative Research Centre Program (CRCP). We also acknowledge the financial and in‐kind support of the project participants. This research has also been supported in part by funding received from the National Health and Medical Research Council’s Centre of Research Excellence funding opportunity [GNT2015611].

## Conflicts of Interest

The authors declare no conflicts of interest.

## Data Availability

Data sharing not applicable to this article as required permissions to share the data were not obtained from participants.
